# Supplementary material for: Low-dose sodium-glucose cotransporter 2 inhibitor ameliorates ischemic brain injury in mice through pericyte protection without glucose-lowering effects
Source: Commun Biol. 2022 Jul 2;5:653. doi: 10.1038/s42003-022-03605-4 (PMC9250510; doi:10.1038/s42003-022-03605-4)
Supplement: Supplementary file 2 — Supplementary Information [file 42003_2022_3605_MOESM2_ESM.pdf]

Low-dose sodium-glucose cotransporter 2 inhibitor ameliorates ischemic brain injury in mice through pericyte protection without glucose-lowering effects

Masamitsu Takashima[1], Kuniyuki Nakamura[1*], Takuya Kiyohara[1], Yoshinobu Wakisaka[1], Masaoki Hidaka[1], Hayato Takaki[1], Kei Yamanaka[1], Tomoya Shibahara[1], Masanori Wakisaka[2], Tetsuro Ago[1], and Takanari Kitazono[1]

[1]Department of Medicine and Clinical Science, Graduate School of Medical Sciences, Kyushu University, 3-1-1 Maidashi, Higashi-ku, Fukuoka 812-8582, Japan.

[2]Wakisaka Internal Medicine Clinic, 1-24-19 Fujisaki, Sawara-ku, Fukuoka 814-0013, Japan.

*Corresponding author: Kuniyuki Nakamura

E-mail: nakamura.kuniyuki.524@m.kyushu-u.ac.jp

Supplementary Figures

Supplementary Figure 1. Intraperitoneal glucose tolerance test and neurological function in mice administered with a low dose of luseogliflozin.

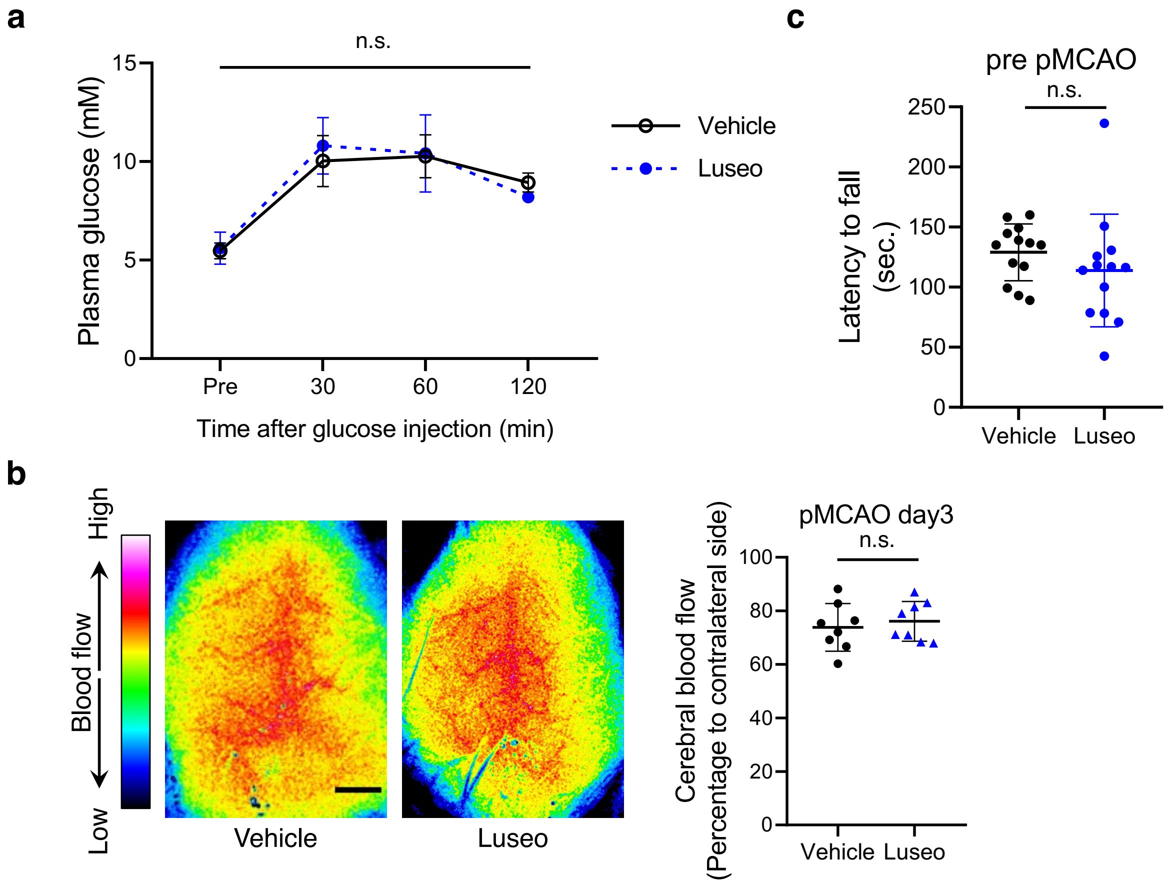

a The mice were intraperitoneally injected with 1 g/kg of D-glucose after overnight fasting, and the plasma glucose levels were measured at 30, 60, and 120 min after glucose injection in vehicle- and luseogliflozin (Luseo)-treated mice (n = 5 mice). Data are presented as mean values ± SD. **b** Cerebral blood flow under the cortical surface was assessed using laser speckle flowmetry at 3 days after pMCAO in vehicle- and Luseo-treated mice. Representative images (left, scale bar = 1 mm) and quantification data (right) are shown (n = 8 mice). **c** Neurological function of the mice was assessed using the rotarod test after the administration of Luseo for 2 weeks (n = 13 mice). Data are presented as dot-plots of individual experiments and mean values ± SD. n.s. (not significant) $P > 0.05$ by unpaired t-test.

Luseo: luseogliflozin, pMCAO: permanent middle cerebral artery occlusion.

Supplementary Figure 2. SGLT2 expression in the kidney and upregulation of SGLT2 in pericytes after pMCAO.

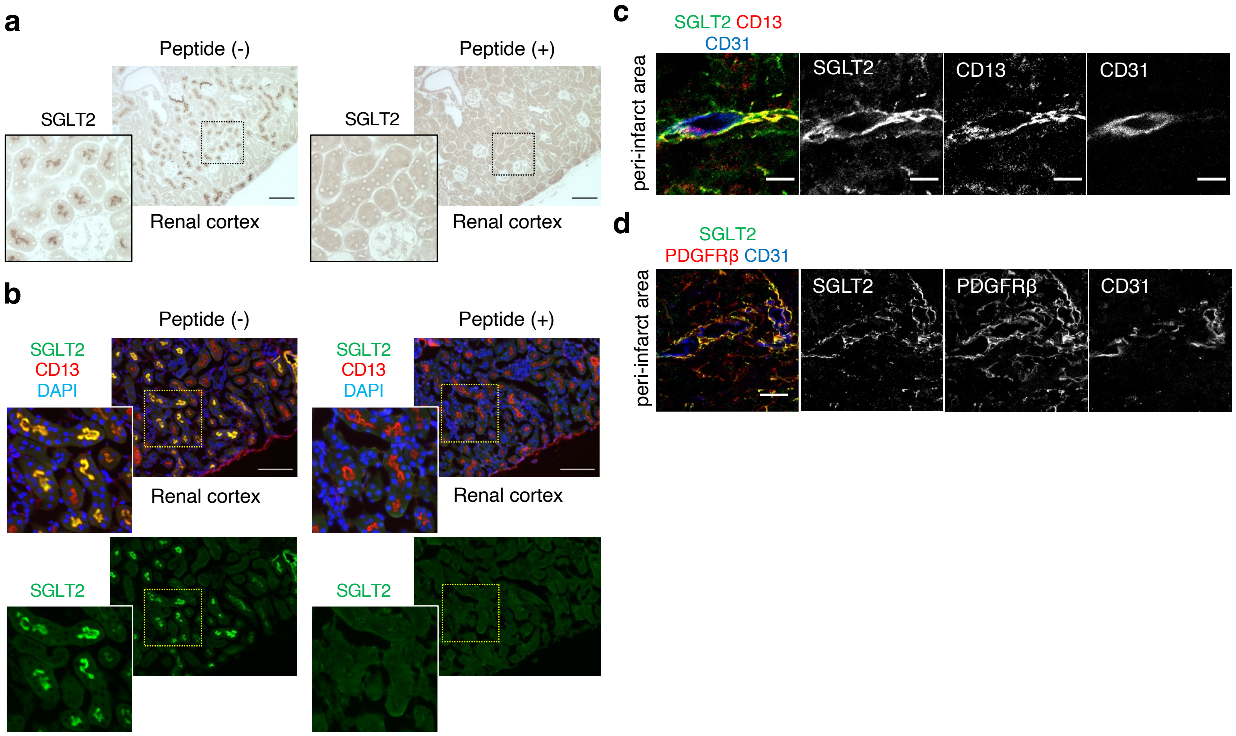

a, b Anti-SGLT2 antibody absorption tests. **(a)** Representative images of immunohistochemical absorption test for anti-SGLT2 antibody in the renal cortex of a C57BL/6JJcl mouse, using the immunizing human SGLT2 peptide (scale bar = 100 μm). The insets indicate a higher magnification of the boxed region. **(b)** Representative images of immunofluorescence staining for SGLT2 (green) and CD13 (red) in the renal cortex of a C57BL/6JJcl mouse after antibody absorption with the immunizing human SGLT2 peptide (scale bar = 100 μm). The insets indicate a higher magnification of the boxed region. **c, d** Adult C57BL/6JJcl mice were subjected to pMCAO. **(d)** Representative magnified images of immunofluorescence for SGLT2 (green), CD13 (red), and CD31 (blue) in microvessels in a peri-infarct area 7 days after pMCAO (scale bar = 10 μm). **(e)** Representative magnified images of immunofluorescence for SGLT2 (green), PDGFRβ (red), and CD31 (blue) in microvessels in a peri-infarct area 7 days after pMCAO (scale bar = 20 μm).

SGLT2: sodium-glucose cotransporter 2.

Supplementary Figure 3. Expression of glucose transporters in brain pericytes.

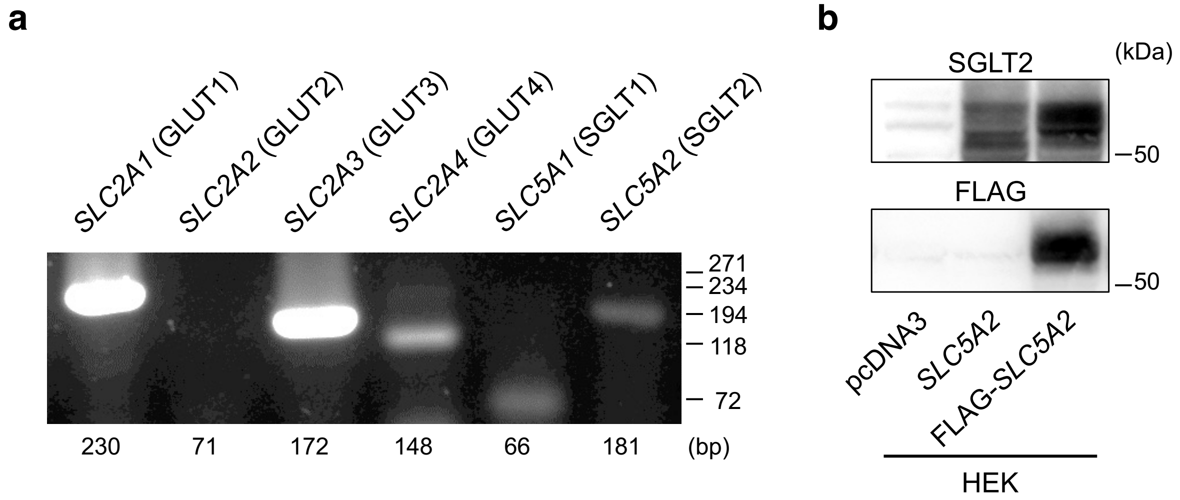

a Representative example of *SLC2A1* (GLUT1), *SLC2A2* (GLUT2), *SLC2A3* (GLUT3), *SLC2A4* (GLUT4), *SLC5A1* (SGLT1), and *SLC5A2* (SGLT2) expression in cultured pericytes assessed by RT-PCR. **b** Representative immunoblotting for SGLT2 and FLAG in HEK-293T cells after transfection with pcDNA3, pcDNA3-*SLC5A2*, or pcDNA3-FLAG-*SLC5A2*.

Supplementary Figure 4. Expression of SGLT2 in pericytes cultured in a medium with high glucose level.

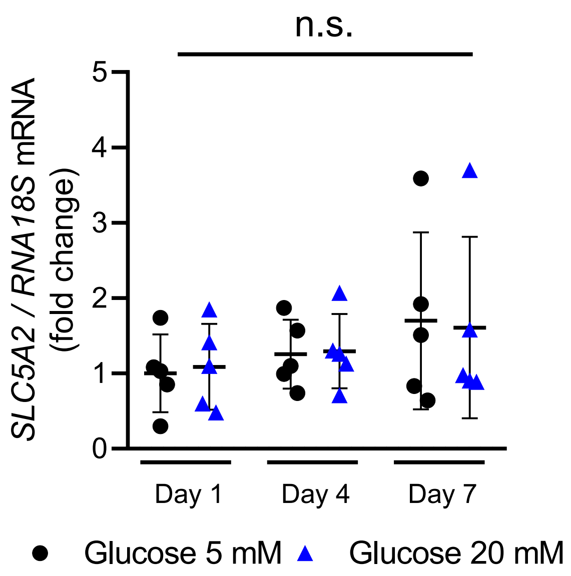

Cultured pericytes were treated with Dulbecco's modified eagle medium containing different glucose levels (5 or 20 mmol/L) for 24, 96, and 168 h. The mRNA expression level of *SLC5A2* (SGLT2) was quantified using qPCR and normalized to that of *RNA18S* ($n = 5$). Data are presented as dot-plots of individual experiments and mean values ± SD. n.s. (not significant) $P > 0.05$ by One-way ANOVA followed by Bonferroni's post hoc test.

Supplementary Figure 5. Luseogliflozin activates mitochondrial biogenesis in brain pericytes.

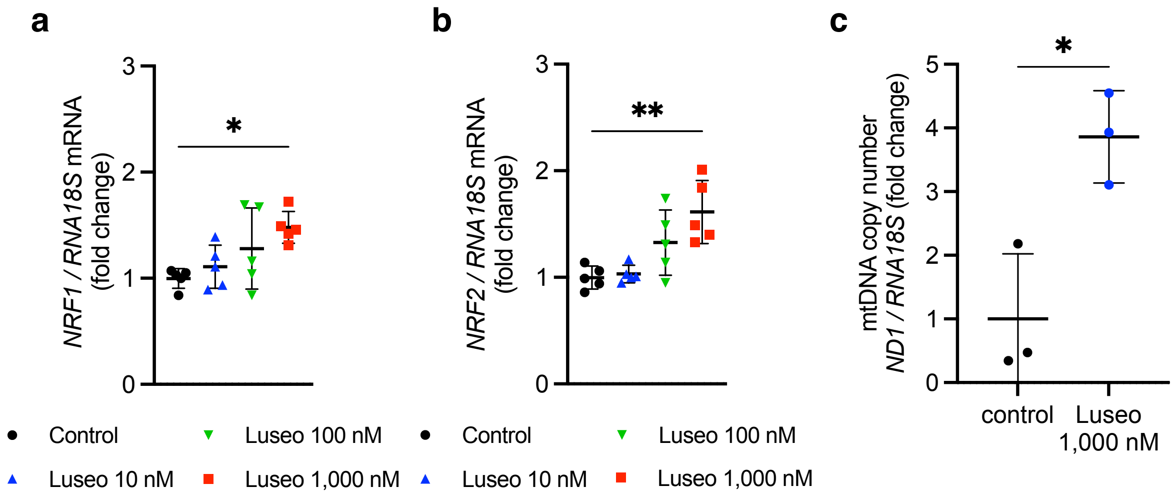

a,b Cultured brain pericytes were treated with 0, 10, 100, or 1,000 nmol/L luseogliflozin (Luseo) for 24 h. The expression levels of *NRF1* **(a)** and *NRF2* **(b)** were quantified using qPCR (n = 5). **c** Cultured pericytes were treated with 0 or 1,000 nmol/L luseogliflozin for 24 h. The expression levels of *ND1* (mtDNA) were quantified using qPCR and normalized to that of *RNA18S* (nDNA) (n = 3). Data are presented as dot-plots of individual experiments and mean values ± SD. *P < 0.05, **P < 0.01 by One-way ANOVA followed by Bonferroni's post hoc test.

Supplementary Figure 6. Uncropped scans of PCR gels and immunoblotting.

Fig. 3a

SLC5A2 *RNA18S*

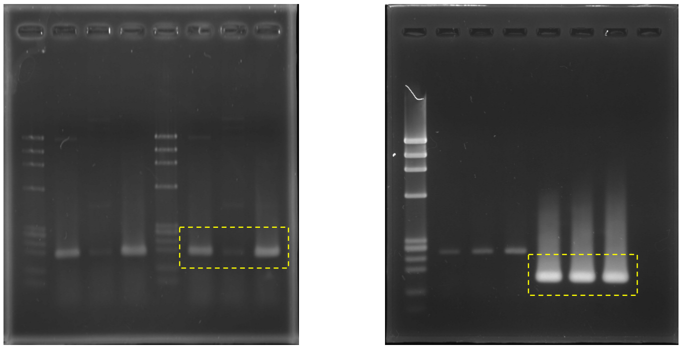

Fig. 3c

SGLT2

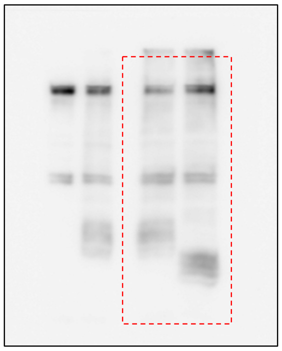

Fig. 3b

SGLT2 CD13 β-actin

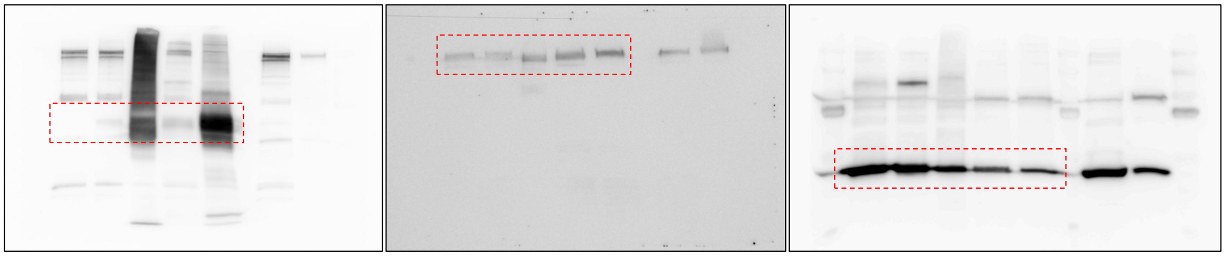

Fig. 5a

TFAM PGC-1α pAMPKα^{T172}

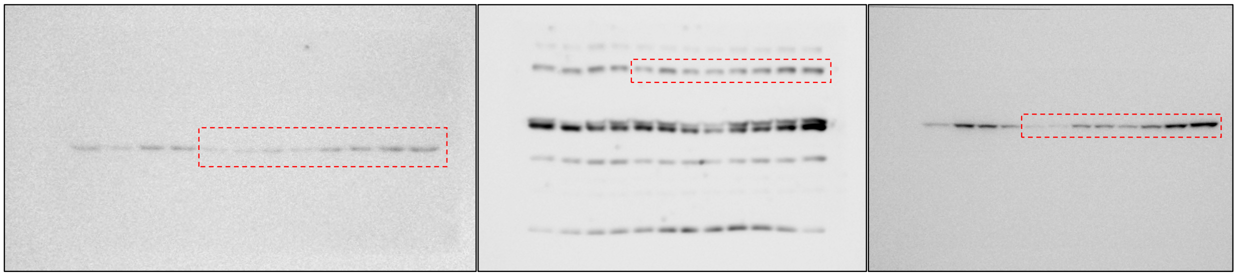

AMPKα β-actin

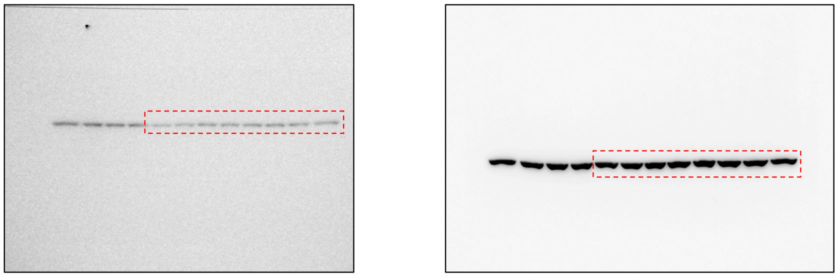

Fig. 5e

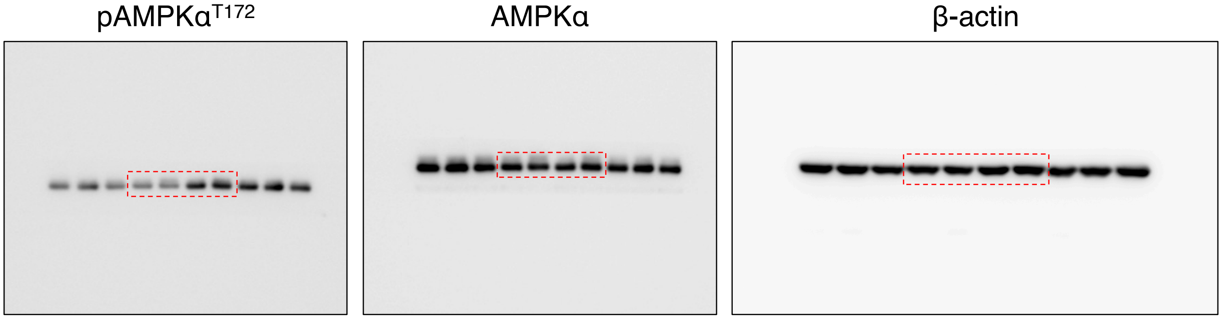

Supplementary Fig. 3a

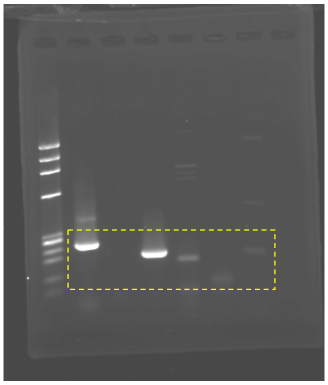

Supplementary Fig. 3b

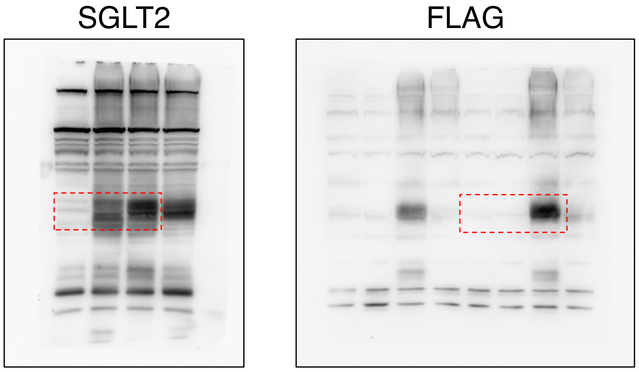

Supplementary Tables

Supplementary Table 1. Physiological data of vehicle- or luseogliflozin-treated mice before and after pMCAO.

	Body weight (g)	SBP (mmHg)	DBP (mmHg)	HR (bpm)
Vehicle	24.1 ± 0.8	108.8 ± 10.5	65.9 ± 7.5	504.6 ± 33.1
Luseogliflozin	24.4 ± 0.8	108.1 ± 3.1	58.1 ± 9.0	555.3 ± 101.9
Vehicle-pMCAO day 3	21.5 ± 1.4	112.4 ± 14.4	51.8 ± 14.9	421.7 ± 41.6
Luseogliflozin-pMCAO day 3	21.9 ± 0.8	109.9 ± 12.0	54.4 ± 15.4	453.1 ± 33.3

Data are presented as the mean ± SD. There was no significant difference between vehicle- and luseogliflozin-treated mice by unpaired *t*-test ($n = 5$, each group).

pMCAO: permanent middle cerebral artery occlusion, SBP: systolic blood pressure, DBP: diastolic blood pressure, HR: heart rate.

Supplementary Table 2. Baseline casual plasma glucose and casual urinary glucose levels in mice.

	Casual plasma glucose (mmol/L)	Casual urinary glucose (mmol/L)
Vehicle	11.2 ± 2.2	7.5 ± 3.4
Luseogliflozin	12.0 ± 1.3	13.0 ± 1.6*
Vehicle-pMCAO day 3	8.5 ± 1.1	10.7 ± 3.5
Luseogliflozin-pMCAO day 3	8.6 ± 1.0	7.6 ± 1.9

Data are presented as the mean ± SD. Mice were administered with vehicle or a low dose of luseogliflozin for 2 weeks, and the casual plasma and urinary glucose levels were measured at baseline and 3 days after pMCAO. *$P < 0.05$ (vehicle vs. luseogliflozin), unpaired *t*-test.

Supplementary Table 3. Modified neurological deficit score.

Normal motor function without any apparent deficit	0
Flexion of the contralateral forelimb when suspended vertically by the tail	1
Spontaneous movement in all directions at rest and circling to the contralateral side when pulled by the tail	2
Spontaneous circling to the contralateral side	3
Loss of righting reflex and decreased resistance to lateral push	4
No spontaneous motor activity	5
