# Supplementary material for: Low-dose sodium-glucose cotransporter 2 inhibitor ameliorates ischemic brain injury in mice through pericyte protection without glucose-lowering effects
Source: Commun Biol. 2022 Jul 2;5:653. doi: 10.1038/s42003-022-03605-4 (PMC9250510; doi:10.1038/s42003-022-03605-4)
Supplement: Supplementary file 3 — Description of Additional Supplementary Files [file 42003_2022_3605_MOESM3_ESM.pdf]

Description of Additional Supplementary Files

File name: Supplementary Data 1
Description: The source data for Figures 1-5, Supplementary Figures 1, 4, and 5, and Supplementary Tables 1 and 2.
