# Supplementary material for: Low-dose sodium-glucose cotransporter 2 inhibitor ameliorates ischemic brain injury in mice through pericyte protection without glucose-lowering effects
Source: Commun Biol. 2022 Jul 2;5:653. doi: 10.1038/s42003-022-03605-4 (PMC9250510; doi:10.1038/s42003-022-03605-4)
Supplement: Supplementary file 5 — Reporting Summary [file 42003_2022_3605_MOESM5_ESM.pdf]

nature portfolio

Corresponding author(s): Kuniyuki Nakamura

Last updated by author(s): May 27, 2022

Reporting Summary

Nature Portfolio wishes to improve the reproducibility of the work that we publish. This form provides structure for consistency and transparency in reporting. For further information on Nature Portfolio policies, see our Editorial Policies and the Editorial Policy Checklist.

Statistics

For all statistical analyses, confirm that the following items are present in the figure legend, table legend, main text, or Methods section.

n/a	Confirmed	
☐	☒	The exact sample size (*n*) for each experimental group/condition, given as a discrete number and unit of measurement
☐	☒	A statement on whether measurements were taken from distinct samples or whether the same sample was measured repeatedly
☐	☒	The statistical test(s) used AND whether they are one- or two-sided<br>*Only common tests should be described solely by name; describe more complex techniques in the Methods section.*
☒	☐	A description of all covariates tested
☐	☒	A description of any assumptions or corrections, such as tests of normality and adjustment for multiple comparisons
☐	☒	A full description of the statistical parameters including central tendency (e.g. means) or other basic estimates (e.g. regression coefficient) AND variation (e.g. standard deviation) or associated estimates of uncertainty (e.g. confidence intervals)
☐	☒	For null hypothesis testing, the test statistic (e.g. *F*, *t*, *r*) with confidence intervals, effect sizes, degrees of freedom and *P* value noted<br>*Give P values as exact values whenever suitable.*
☒	☐	For Bayesian analysis, information on the choice of priors and Markov chain Monte Carlo settings
☒	☐	For hierarchical and complex designs, identification of the appropriate level for tests and full reporting of outcomes
☒	☐	Estimates of effect sizes (e.g. Cohen's *d*, Pearson's *r*), indicating how they were calculated

Our web collection on statistics for biologists contains articles on many of the points above.

Software and code

Policy information about availability of computer code

Data collection	Microscopy images were collected using a confocal microscope Nikon A1R (Nikon) or a fluorescence microscope BIOREVO BZ-9000 microscope (Keyence Corporation).<br>Blood pressure and heart rate of mice were measured using a noninvasive tail-cuff BP system (BP-2000, Visitech Systems).<br>The glucose level was measured using FreeStyle Precision Neo (Abbott).<br>Relative cerebral blood flow was determined by laser speckle flowmetry (Omegazone OZ-2, Omegawave Inc.).<br>Absorbance and fluorescence intensity were measured using a microplate reader (ARVO X4, Perkin Elmer).<br>Quantitative PCR was performed using a LightCycler (Roche Diagnostics GmbH).<br>Bands of PCR and immunoblotting were detected in a luminescent image analyzer (LAS-3000, Fujifilm)
Data analysis	Acquired images were analyzed using ImageJ software (NIH). GraphPad Prism Version 8.0 (GraphPad Software) was used for all analyses.

For manuscripts utilizing custom algorithms or software that are central to the research but not yet described in published literature, software must be made available to editors and reviewers. We strongly encourage code deposition in a community repository (e.g. GitHub). See the Nature Portfolio guidelines for submitting code & software for further information.

Data

Policy information about availability of data

All manuscripts must include a data availability statement. This statement should provide the following information, where applicable:

- Accession codes, unique identifiers, or web links for publicly available datasets
- A description of any restrictions on data availability
- For clinical datasets or third party data, please ensure that the statement adheres to our policy

The experimentally data that support the findings of this study are available within the article and its Supplementary figure files or from the corresponding authors upon reasonable request.

Field-specific reporting

Please select the one below that is the best fit for your research. If you are not sure, read the appropriate sections before making your selection.

☒ Life sciences ☐ Behavioural & social sciences ☐ Ecological, evolutionary & environmental sciences

For a reference copy of the document with all sections, see nature.com/documents/nr-reporting-summary-flat.pdf

Life sciences study design

All studies must disclose on these points even when the disclosure is negative.

Sample size	Sample size was estimated based on previous experiments performed in our lab (Shibahara et al. Stroke 2020).
Data exclusions	No data were excluded from this study.
Replication	All experimental findings were reliably reproduced by replicating the experiments in both mice and cellular systems.
Randomization	Experimental and control mice were randomly chosen from littermates. Random ROIs were selected for imaging analysis.
Blinding	Investigators were blinded when performing for the quantification of images.

Reporting for specific materials, systems and methods

We require information from authors about some types of materials, experimental systems and methods used in many studies. Here, indicate whether each material, system or method listed is relevant to your study. If you are not sure if a list item applies to your research, read the appropriate section before selecting a response.

Materials & experimental systems

n/a	Involved in the study	
☐	☒	Antibodies
☐	☒	Eukaryotic cell lines
☒	☐	Palaeontology and archaeology
☐	☒	Animals and other organisms
☒	☐	Human research participants
☒	☐	Clinical data
☒	☐	Dual use research of concern

Methods

n/a	Involved in the study	
☒	☐	ChIP-seq
☒	☐	Flow cytometry
☒	☐	MRI-based neuroimaging

Antibodies

Antibodies used

Primary Antibodies:
Anti-MAP2 (Mouse monoclonal, #M4403, Sigma-Aldrich)
Anti-CD31 (Rat monoclonal, #550274, BD Biosciences)
Anti-CD31 (Rat monoclonal, #ab56299, Abcam)
Anti-CD13 (Goat polyclonal, #AF2335, R&D Systems)
Anti-PDGFRβ (Goat polyclonal, #AF1042, R&D Systems)
Anti-SGLT2 (Rabbit polyclonal, #ab85626, Abcam)
Anti-SGLT2 (Rabbit polyclonal, #14210, CST)
Anti-TFAM (Rabbit monoclonal, #8076S, CST)
Anti-phosphorylated-AMPKα (Thr172) (Rabbit monoclonal, #2535, CST)
Anti-AMPKα (Rabbit monoclonal, #5831, CST)

	Anti-PGC-1α (Rabbit polyclonal, #AB3242, Millipore)<br>Anti-β-actin (Mouse monoclonal, #A5441, Sigma-Aldrich)<br>Anti-FLAG M2 (Mouse monoclonal, #F3165, Sigma)<br>Secondary antibodies:<br>Alexa-488-conjugated anti-rat IgG (#A21208, Invitrogen)<br>Alexa-568-conjugated anti-rabbit IgG (#A10042, Invitrogen)<br>Alexa-647-conjugated anti-goat IgG (#A21447, Invitrogen)<br>HRP-linked anti-mouse IgG (#7076, CST)<br>HRP-linked anti-rabbit IgG (#7074, CST)
Validation	We used sections of the renal cortex of C57BL/6JJcl mice as a positive control to confirm the specificity of the anti-SGLT2 antibody (Supplementary Fig. 2a, 2b).<br><br>Anti-MAP2 (https://www.sigmaaldrich.com/US/en/product/sigma/m4403)<br>Anti-CD31 (https://www.bdbiosciences.com/en-us/products/reagents/flow-cytometry-reagents/research-reagents/single-color-antibodies-ruo/purified-rat-anti-mouse-cd31.550274)<br>Anti-CD31 (https://www.abcam.co.jp/cd31-antibody-rm0032-1d12-bsa-and-azide-free-ab56299.html)<br>Anti-CD13 (https://www.rndsystems.com/products/mouse-aminopeptidase-n-cd13-antibody_af2335)<br>Anti-PDGFRβ (https://www.rndsystems.com/products/mouse-pdgf-rbeta-antibody_af1042)<br>Anti-TFAM (https://www.cellsignal.com/products/primary-antibodies/tfam-d5c8-rabbit-mab/8076)<br>Anti-phosphorylated-AMPKα (Thr172) (https://www.cellsignal.jp/products/primary-antibodies/phospho-ampka-thr172-40h9-rabbit-mab/2535)<br>Anti-AMPKα (https://www.cellsignal.jp/products/primary-antibodies/ampka-d5a2-rabbit-mab/5831)<br>Anti-PGC-1α (https://www.merckmillipore.com/JP/ja/product/Anti-PGC-1-Antibody,MM_NF-AB3242)<br>Anti-β-actin (https://www.sigmaaldrich.com/US/en/product/sigma/a5441)<br>Anti-SGLT2 (https://www.abcam.co.jp/sglt2-antibody-ab85626.html)<br>Anti-SGLT2 (https://www.cellsignal.jp/products/primary-antibodies/sglt2-antibody/14210)<br>Anti-FLAG (https://www.sigmaaldrich.com/JP/ja/product/sigma/f3165)

Eukaryotic cell lines

Policy information about cell lines

Cell line source(s)	HEK-293T cells and HK-2 cells were obtained from ATCC (American Type Culture Collection).
Authentication	Authentication was done by ATCC as per internal quality control procedure. No further authentication was performed.
Mycoplasma contamination	These cell lines were negative for mycoplasma.
Commonly misidentified lines<br>(See ICLAC register)	These cell lines are not known to be cross-contaminated or misidentified, and are not listed in the Register of Misidentified Cell Lines International Cell Line Authentication Committee (ICLAC).

Animals and other organisms

Policy information about studies involving animals; ARRIVE guidelines recommended for reporting animal research

Laboratory animals	C57BL/6JJcl mice were purchased from CLEA Japan (Tokyo, Japan). Male mice aged 8–11 weeks were used in the experiments.
Wild animals	No wild animals were used.
Field-collected samples	No field-collected samples were used.
Ethics oversight	The Animal Care and Use Review Committee of Kyushu University approved the animal experimental protocol (protocol number A20-107-1).

Note that full information on the approval of the study protocol must also be provided in the manuscript.
